# Increasing recognition of statin-associated anti-HMGCR antibody–positive autoimmune myopathy: a single-center retrospective study

**DOI:** 10.1007/s10067-026-08136-5

**Published:** 2026-04-29

**Authors:** Mikko Mali, Laura Pirilä, Manu Jokela, Tero Pääkkö, Markku Mali

**Affiliations:** 1 Faculty of Medicine, Oulu University, Oulu, Finland; 2 Department of Rheumatology, Turku University Hospital, University of Turku, PO Box 52, Turku, 20521 Finland; 3 Department of Neurology, Neurocenter, Turku University Hospital, University of Turku, Turku, Finland; 4 Department of Neurology, Neuromuscular Center, Tampere University Hospital, Tampere, Finland; 5 Department of Rheumatology, Oulu University Hospital, Oulu, Finland

**Keywords:** Anti-HMGCR-Ab, Autoimmune diseases, Autoimmune myopathy, Myositis, Rituximab

## Abstract

**Objective:**

The use of statins, which are inhibitors of the 3-hydroxy-3-methylglutaryl-coenzyme A reductase (HMGCR) enzyme, may in rare cases be associated with immune-mediated necrotizing myopathy. This condition is characterized by the presence of autoantibodies directed against the HMGCR enzyme. Previously considered very rare, the incidence of this myopathy diagnosis has markedly increased in our rheumatology unit over the past years.

**Methods:**

We conducted a retrospective analysis of 13 patients diagnosed with statin-associated anti-HMGCR antibody (Ab)-positive immune-mediated necrotizing myopathy (anti-HMGCR-IMNM) over the 5-year period (June 2020–May 2025) at the Turku University Hospital’s rheumatology department serving 490,000 inhabitants.

**Results:**

Patients presented with elevated creatine kinase (CK) levels, positive anti-HMGCR-Ab, proximal muscle weakness, and the initial symptom was usually difficulty walking. No other myositis autoantibodies were detected and extra-myopathic symptoms were rare. Most patients responded to immunosuppressive therapy including glucocorticoids, methotrexate or azathioprine, and rituximab with a more severe disease course. Disease severity ranged from mild to fatal. The incidence of statin-associated anti-HMGCR-IMNM was 5.3 cases per million per year, approximately 2.75 per 100,000 statin users per year, higher than previously reported.

**Conclusion:**

Statin-associated anti-HMGCR-IMNM is a rare but increasingly recognized condition requiring early diagnosis and immunosuppressive treatment. The clinical presentation is highly variable. Treatment and its intensity must be tailored individually, taking into account the patient’s comorbidities and treatment related risks.

**Table:** 

**Key Points** • *Anti-HMGCR antibody–positive immune-mediated necrotizing myopathy (anti-HMGCR-IMNM) may be triggered by statins, one of the most commonly prescribed classes of medications. Because delayed diagnosis may lead to significant morbidity, it is important that clinicians are familiar with this rare myopathic disorder.* • *Previously, statin-associated anti-HMGCR-IMNM was considered a very rare diagnosis. However, the incidence of this diagnosis appears to have increased markedly over recent years.* • *The apparent increase in diagnoses may be attributable to the growing use of statins; additionally, improved recognition and awareness of the disease may also partially explain this rise.* • *Rituximab may be an alternative in selected cases where the disease course is neither rapidly progressive nor life-threatening, although further research is needed.*

## Introduction

The benefits of statins in the management of cardiovascular disease far outweigh the muscle-related adverse effects associated with their use. In very rare cases, statin therapy may be linked to an immune-mediated necrotizing myopathy (IMNM), with an estimated incidence of 2.0–2.4 cases per 100,000 statin users per year [1]. Patients with this condition exhibit antibodies (Ab) that target the pharmacological site of statins, 3-hydroxy-3-methylglutaryl-coenzyme A reductase (HMGCR). Unlike the toxic statin myopathy associated with the initiation of statin therapy, affecting approximately 1 per 10,000 statin users per year [2], IMNM does not usually resolve after discontinuation of the statin. Statin-associated anti-HMGCR-Ab-positive IMNM (anti-HMGCR-IMNM) is frequently progressive, subacute, and severe, necessitating immunosuppressive treatment. A more chronic form of the disease is also recognized, in which symptoms resemble limb-girdle muscular dystrophy (LGMD) [3–5].

The reported annual incidence of anti-HMGCR-IMNM ranges from 0.9 to 2.9 cases per million population [1, 4–6]. Statins may contribute to disease onset through an immunological mechanism or act as one of several risk factors [6]. The condition also occurs without statin exposure and has been described in children, leading some to suggest that statin therapy may be merely an apatogenic bystander [7]. Statin-independent disease is more common in younger patients and in Asian populations, where extramuscular manifestations are more frequent and the disease tends to be more severe and treatment-resistant [4–6]. In a recent Australian and UK cohort, only 7.5% (8/109) of patients had no history of statin exposure [1].

Given that anti-HMGCR-IMNM can be triggered by one of the most commonly prescribed medications and that delayed diagnosis may lead to serious outcomes, it is important for clinicians to be familiar with this rare muscle disease.

## Materials and methods

We compiled all cases of statin-associated anti-HMGCR-IMNM diagnosed over a 5-year period (June 2020–May 2025) at the Adult Rheumatology Unit of Turku University Hospital. The numbers of cases by study year were 4, 0, 3, 1, and 5, respectively, yielding a total of 13 cases. Treatment data were obtained up to the end of June 2025.

Anti-HMGCR-Ab test was a commercial ELISA test (INOVA QUANTA Lite HMGCR ELISA, San Diego, CA, USA). Anti-HMGCR-Ab testing has been available at Turku University Hospital Laboratories since 2015 and has been implemented in clinical practice in our unit since 2016 [8]. The date of the first positive anti-HMGCR antibody test (> 20 U/mL) was defined as the time of anti-HMGCR-IMNM diagnosis.

Myositis autoantibodies were studied using myositis line-blot immunoassay (LIA; EUROLINE myositis line blot, Autoimmune Inflammatory Myopathies 16 Ag, Euroimmun, Lübeck, Germany). The LIA assay included 12 myositis autoantibodies anti-Mi-α, anti-Mi-β, anti-melanoma differentiation-associated protein 5 (MDA5), anti-transcription intermediary factor 1 (TIF1)-γ, anti-nuclear matrix protein 2 (NXP2), anti-small ubiquitin-like modifier-1 activating enzyme (SAE1), anti-histidyl transfer ribonucleic acid (tRNA) synthetase (Jo-1), anti-threonyl-tRNA synthetase (PL-7), anti-alanyl-tRNA synthetase (PL-12), anti-glycyl-tRNA synthetase (EJ), anti-isoleucyl-tRNA synthetase (OJ), anti-signal recognition particle (SRP), and four myositis-associated autoantibodies anti-polymyositis (PM)-Scl-100, anti-PM-Scl-75, anti-Ku72/86, and anti-Ro52.

Electroneuromyography (ENMG) and muscle biopsies were performed on all but one patient, who recovered rapidly after stopping the statin and were no longer considered necessary.

Magnetic resonance imaging (MRI) of the muscles was performed on all but one patient who had magnetic metal in the imaging area.

As core set measures of disease activity defined by the International Myositis Assessment and Clinical Trial Group were not reliably documented for patients, modified outcomes were used, like time of normalization of creatine kinase (CK) values from diagnosis. In addition, manual muscle testing (MMT8) scores at baseline and follow-up were available for nine out of 13 patients, and health assessment questionnaire (HAQ) data were available for 11 out of 13 patients [9, 10]. Baseline MMT8 assessment was missing for four patients, baseline HAQ data for one patient, and follow-up HAQ data for one patient. Follow-up values closest to 12 months (range 3–14 months) were used. Four patients had follow-up data available only at 3–6 months. In three of these patients, the disease duration at the time of data collection was less than 1 year, and one patient died due to a cardiac event 5 months after diagnosis.

Rituximab therapy was initiated according to a standard induction protocol consisting of two 1000-mg infusions administered 2 weeks apart. In two patients, induction therapy alone was sufficient, whereas two additional patients received maintenance rituximab (500–1000 mg) administered as single infusions at approximately 6-month intervals, for a total of two additional doses (Table 2).

When estimating the incidence of anti-HMGCR-IMNM among statin users, we used drug utilization data from 2023, which corresponds to the midpoint of our study cohort. According to the Finnish Medicines Statistics, the Defined Daily Dose of statins in 2023 was 193.40 per 1000 inhabitants per year [11].

In a patient with an LGMD-like phenotype, whole-exome sequencing was performed using a neuromuscular disease gene panel comprising 496 genes; no pathogenic variants were identified.

The study protocol adhered to the principles of the Declaration of Helsinki. This was a non-interventional retrospective study without direct patient contact. According to Finnish legislation, no patient consent or ethical committee approval was needed. Permissions for the study were obtained from the Wellbeing Service County of Southwest Finland (VARHA Research Permit T1148/2025,VARHA Information Permit 2025–1157-TL).

## Results

A total of 13 patients were identified (4 men and 9 women). The mean age at diagnosis was 74.7 years (range 62–85 years). Men were younger (mean 66.3 years) than women (mean 78.4 years). At the time of diagnosis, 11 patients were receiving atorvastatin (20–80 mg) and two were on rosuvastatin (5–20 mg). The median duration of statin therapy prior to diagnosis was 4 years (range 1.2–18 years), and the median duration of muscle symptoms before diagnosis was 6 months (range 3–14 months) (Table 1). The most common initial symptom was difficulty walking.

**Table 1:** Demographic, clinical, and treatment-related characteristics of patients

	***n*** (%), mean or median (range)
Sex, male/female	4/9
Age at diagnosis, mean (range), years:	74.7 (62–85)
*In males*	66.3 (62–74)
*In females*	78.4 (69–85)
Statin use, *n* (%)	13 (100)
*Atorvastatin*	11 (85)
*Rosuvastatin*	2 (15)
Duration of statin use, median (range), years	4 (1.17–18)
CK level at diagnosis, median (range), U/L	5658 (1052–15,422)
Time to CK normalization after treatment initiation, median (range), days	185 (44–540)
Anti-HMGCR-Ab concentration at diagnosis, median (range), U/mL	170 (41– > 200)*
Onset of muscle symptoms before diagnosis, median (range), months:	6 (3–14)
Medication, *n* (%)	
*No pharmacological treatment*	1 (7.7)
*Prednisolone*	12 (92.3)
*Methotrexate*	9 (69.2)
*Azathioprine*	1 (7.7)
*Rituximab*	5 (38.5)
Co-morbidities, *n* (%)	
*Coronary artery disease*	9 (69)
*Hypertension*	11 (85)
*Diabetes mellitus (type 2)*	8 (62)
*Cerebral infarction*	3 (23)
*Transient ischemic attack (TIA)*	2 (15)
Death, *n* (%)	2 (15)**

^*^Upper limit of the test: 200 U/mL. Patients with concentrations above 200 *n* = 5

^**^One patient’s cause of death was a cardiac event after having recovered well from myopathy; the other died within 4 weeks of diagnosis as a consequence of anti-HMGCR-IMNM

Comorbidities were common, like coronary artery disease (*n* = 9, 69%), hypertension (*n* = 11, 85%), type 2 diabetes mellitus (*n* = 8, 62%), cerebral infarction (*n* = 3, 23%), and transient ischemic attack (*n* = 2, 15%) (Table 1).

Electroneuromyography (ENMG) was performed in 12 patients and revealed proximal myopathic changes. Muscle biopsies (*n* = 12) showed findings consistent with IMNM [12], including scattered necrotic muscle fibers with minimal inflammatory infiltrates. Magnetic resonance imaging (MRI) of the muscles (*n* = 12) typically demonstrated edema, particularly in the thigh and pelvic muscles. One patient exhibited a limb-girdle muscular dystrophy (LGMD)-like pattern with fatty infiltration of the shoulder and paraspinal muscles.

The median CK level at diagnosis was 5658 U/L (range 1052–15,422). Anti-HMGCR-Ab levels had a median of 170 U/mL (range 41– > 200), with five patients exceeding the upper detection limit of > 200 U/mL. No other myositis-specific or myositis-associated antibodies were detected in the myositis antibody panel (EUROLINE myositis profile). Two patients had unspecific borderline positive antinuclear antibody titers of 1:320 (< 320), with a homogeneous staining pattern. CK levels normalized (men < 280 U/L, women < 210 U/L) at a median of 185 days (range 44–540) from diagnosis (*n* = 12).

Prednisolone therapy (initial dose 10–80 mg) was initiated in all but one patient (*n* = 12, 92%). One patient also received an initial 3-day pulse of intravenous methylprednisolone (500 mg). Methotrexate was the most commonly used adjunct immunosuppressant (*n* = 9, 69%). One patient received azathioprine (8%), and rituximab was administered to five patients (39%) with more severe disease. Following statin discontinuation, all patients were started on ezetimibe for hypercholesterolemia, and PCSK-9 inhibitor evolocumab (*n* = 8) was added when national reimbursement criteria were met.

**Fig. 1:**
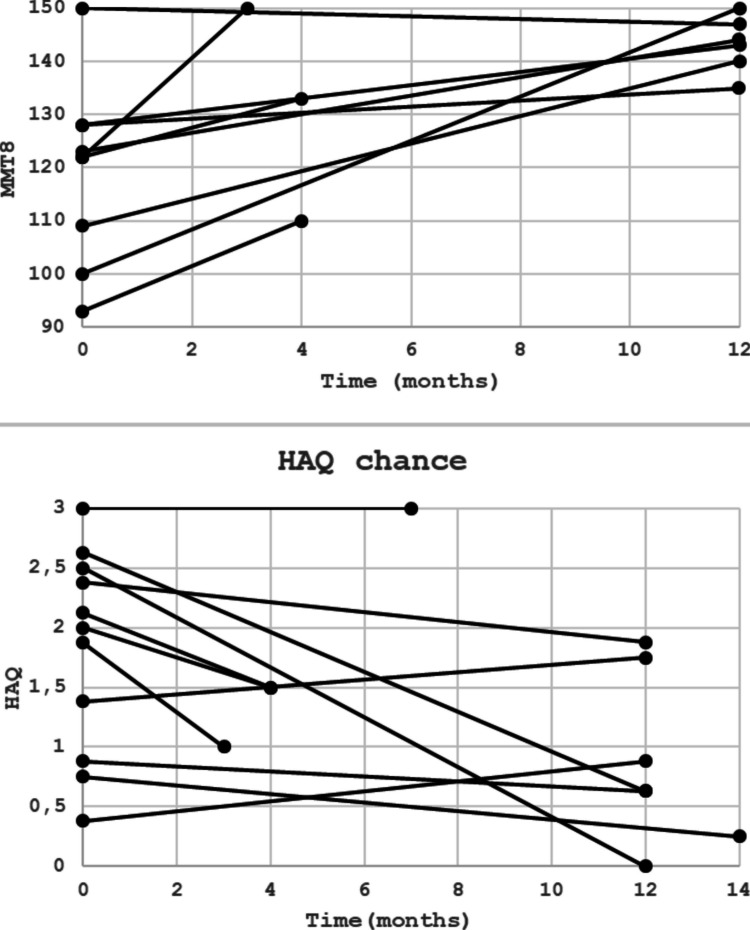
Changes in muscle strength and functional status. Manual muscle testing (bilateral MMT8, maximum 150) scores at baseline and follow-up were available for nine of the 13 patients, and Health Assessment Questionnaire (HAQ) data were available for 11 of patients. Muscle strength improved in eight patients except for the individual who experienced an ischemic stroke during follow-up; this patient also demonstrated a decline in functional capacity. Functional capacity did not improve (HAQ score did not decrease) in the patient with an LGMD-type disease phenotype, whose affected muscles were already extensively replaced by fat at the time of diagnosis. One patient had permanently reduced functional capacity (HAQ 3.0) due to a previous hemiparesis

Manual muscle testing (bilateral MMT8, maximum 150) scores at baseline and follow-up were available for nine patients, and Health Assessment Questionnaire (HAQ) data were available for 11 patients. Functional capacity improved in 8/11 of these individuals. One patient who experienced an ischemic stroke during follow-up demonstrated a slight decline in both muscle strength and functional capacity. Functional capacity did not improve in the patient with an LGMD-type disease phenotype, whose affected muscles were already extensively replaced by fat at the time of diagnosis. One patient had permanently reduced functional capacity due to a previous hemiparesis (Fig. 1).

The two oldest patients represented the extremes of the disease spectrum. In one case (female, 84 years), the condition was identified at an early stage (HMGCR-Ab 41 U/mL, CK 3992 U/L, MRI showing mild changes). This patient recovered rapidly, and CK normalized with statin discontinuation alone, without the need for immunosuppressive therapy. The other patient (female, 85 years) presented with a rash, markedly elevated CK of 10,071 U/L, and anti-HMGCR-Ab > 200 U/mL. Treatment was initiated with prednisolone 60 mg daily, resulting in a CK decrease to approximately 2500 U/L. Rituximab 1000 mg IV was administered, and high-dose intravenous immunoglobulin (IVIg) therapy was planned as the next step. However, IVIg could not be administered; the patient developed rapidly progressive dysphagia and respiratory failure at the follow-up facility and died within 4 weeks of diagnosis.

No other patients in the cohort exhibited dysphagia, rash, or extramuscular symptoms such as Raynaud’s phenomenon, arthritis, or interstitial lung disease. Additionally, no active malignancies were observed during the follow-up period or 3 years before diagnosis of anti-HMGCR-IMNM. One patient died of myocardial infarction 5 months after the diagnosis of anti-HMGCR-IMNM, at a time when he had already recovered well from myopathy and with normalized CK levels.

Four more severely affected patients initiated rituximab therapy 60–101 days after disease onset. These patients achieved normalization of CK levels 123–540 days after diagnosis (Table 2). Their initial CK values ranged from 5658 to 15,422 U/L, and serum anti-HMGCR-Ab levels were 158 U/mL to > 200 U/mL.

**Table 2:** Treatment data and summary of therapeutic responses in four rituximab-treated patients

Age, sex	CK at baseline (U/L)	Anti-HMGCR Ab (U/mL)	Initial pred dose, (mg)	DMARD	RTX started after DG (d)	Pred to 5 mg (d)	CK normalization time (d)	HAQ at baseline	HAQ 1 year	MMT8 at baseline	MMT8 1 year
70, F	**9 460**	**185**	**60**	**AZA 100 mg**	101***	**269**	**540**	**2.38**	**1.88**	**109**	**140**
74, F	**15 422**	** > 200**	60*	**None**	**60**	**321**	**185**	**2.63**	**0.63**	**NA**	**137**
65, M	**7 046**	** > 200**	**40**	**MTX 15 mg/week**	84***	**290**	**472**	**0.88**	**0.63**	**123**	**144**
62, M	**5 658**	**158**	**80**	**MTX 20 mg/week**	**60**	**231**	**123**	**1.88**	1.00^**^	**122**	150^**^

^*^Started with methylprednisolone 500 mg i.v. 3 days following prednisolone

^**^HAQ and MMT8 values 3-month control instead of 1 year

^***^Following induction, single rituximab infusions were administered at 6 and 12 months

*F* female, *M* male, *CK* creatine kinase, *HMGCR* 3-hydroxy-3-methylglutaryl-coenzyme A reductase, *Pred* prednisolone, *DMARD* disease-modifying anti-inflammatory drug, *AZA* azathioprine, *MTX* methotrexate, *RTX* rituximab, *d* day after diagnosis of myopathy, *DG* Diagnosis, *HAQ* Health Assessment Questionnaire, *MMT8* manual muscle test 8, *NA* not available

Table 2 summarizes changes in functional status, assessed using the HAQ index, and muscle strength, evaluated using MMT8, over a 1-year period. For one patient, data was available only at the 3-month time point because disease duration at the time of data collection was less than 1 year. Both functional capacity and muscle strength improved in all four patients.

Anti-HMGCR-Ab levels were measured after rituximab therapy in two patients before the completion of data collection. In both patients, baseline anti-HMGCR-Ab levels exceeded 200 U/mL. One patient (74-year-old woman) received glucocorticoid therapy and induction rituximab only; her anti-HMGCR-Ab level decreased to 18 U/mL at 479 days after diagnosis. In the other patient (65-year-old man), the antibody level decreased to 52 U/mL at 472 days after diagnosis.

**Fig. 2:**
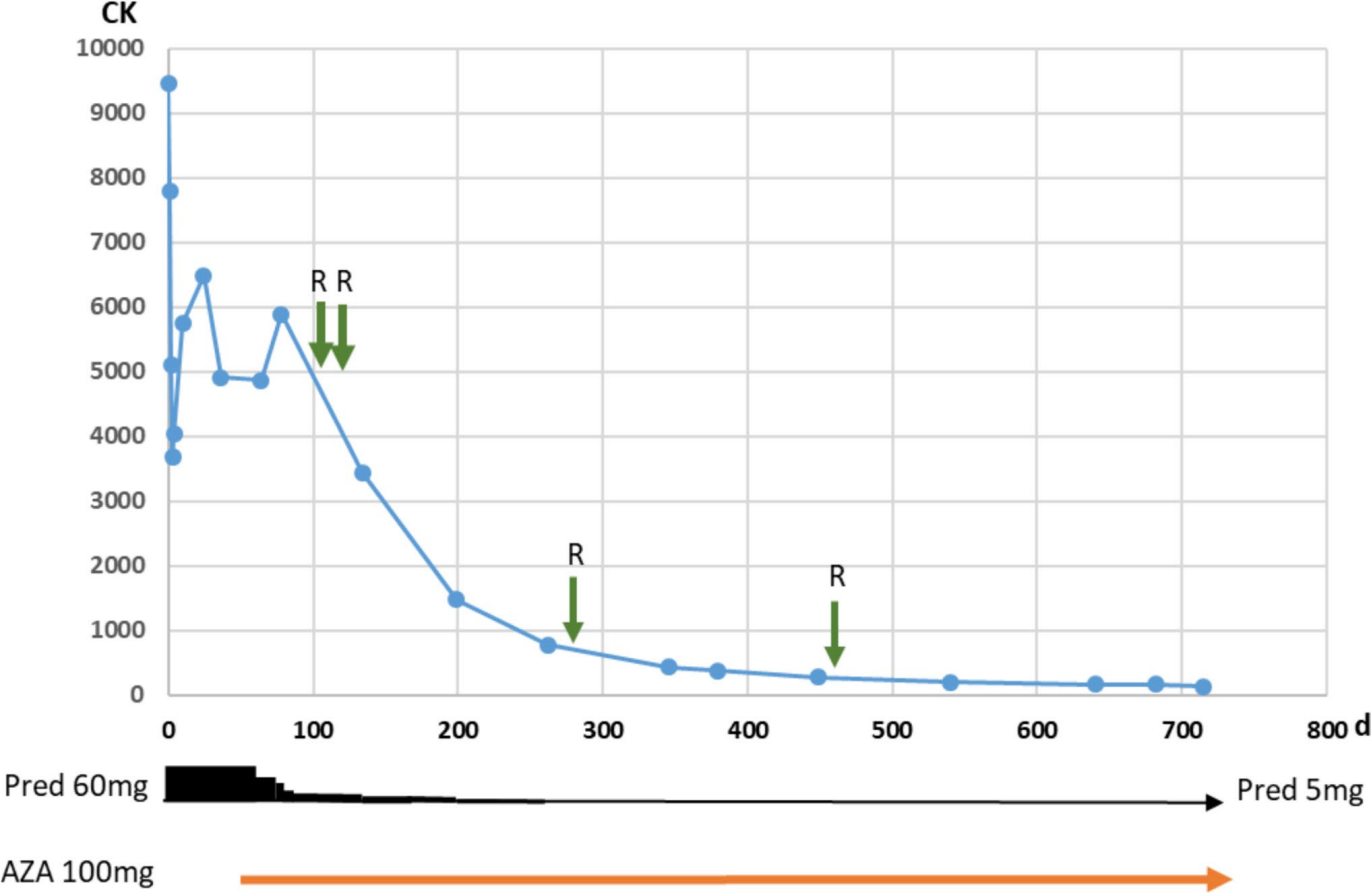
Treatment of a patient (female, 70 years, initial CK 9460 U/L) receiving rituximab and change in creatine kinase values. Pred, prednisolone; AZA, azathioprine; CK, creatine kinase; R, rituximab infusion; bolder down arrow, rituximab 1000 mg; down arrow, rituximab 500 mg; d, days after myopathy diagnosis

Figure 2 illustrates the course of the rituximab-treated patient (female, 70 years, anti-HMGCR-Ab 185 U/mL, initial CK 9460 U/L), in whom the initial decline in CK levels appeared to plateau but subsequently began to decrease gradually following rituximab therapy.

## Discussion

Between June 2020 and April 2025, we diagnosed 13 cases of statin-associated anti-HMGCR-IMNM in a population of 490,000, corresponding to an annual incidence of 5.3 per million, or approximately 2.75 per 100,000 statin users per year based on 2023 Finnish statin consumption data [11]. This exceeds previously reported rates of anti-HMGCR-IMNM from 0.9 to 2.9 cases per million population [1, 4–6]. Small numbers and annual variability may partly explain the difference. In a Finnish study reviewing myositis antibody testing from 2015 to 2020, the frequency of positive anti-HMGCR-Ab findings was only 0.67 per million per year [8]. Before June 2020, the 5-year incidence of anti-HMGCR-IMNM in our department was only 0.8 per million per year. Anti-HMGCR-Ab were first identified in the early 2010 s in patients whose CK levels and muscle weakness persisted despite statin discontinuation [4]. Increased recognition may partially reflect expanding testing and prior underdiagnosis or misclassification as other myopathies in Finland.

The rising incidence may also be influenced by stricter lipid-lowering targets, as statin consumption increased in Finland by nearly 90% between 2016 and 2023 [11]. Anti-HMGCR-IMNM is most commonly observed in individuals over 50 years of age, with a mean onset between 60 and 70 years [3–5]. In our series, men developed the disease at a younger age (mean 66 years) compared to women (mean 78 years). The HLA-DRB1*11:01 allele and possibly other genetic factors have been associated with increased susceptibility [6, 13]. Interestingly, our cohort included identical twin brothers who were diagnosed with IMNM within 3 months of each other [13]. In addition, another patient in the cohort had an older sister who had previously been diagnosed with statin-associated anti-HMGCR-IMNM.

There are no randomized controlled trials comparing treatments for anti-HMGCR-IMNM; current recommendations rely on case series and expert opinion [6, 14, 15]. The European Neuromuscular Centre (ENMC) guidelines recommend initiating therapy with glucocorticoids together with an immunosuppressant (methotrexate, azathioprine, or mycophenolate), with high-dose IVIg (2 g/kg over 2 days, repeated monthly) added as needed. If the response remains inadequate after 6 months, rituximab is suggested [6, 14, 16, 17]. The British Society for Rheumatology guidelines suggest rituximab as an option in autoantibody-positive inflammatory myopathies [18]. In the Bristol cohort, 41.2% of anti-HMGCR-IMNM patients received rituximab [1], and multiple case reports have described favorable outcomes [19]. According to the recently updated treatment recommendations of the Swedish Rheumatology Association [20], rituximab may also be considered for the management of anti-HMGCR-IMNM, and in cases of severe or refractory disease, the use of IVIg is recommended. Rituximab is commonly administered at a dose of 500–1000 mg as two infusions given 2 weeks apart, followed by maintenance infusion every 6 months for up to 2 years when required.

In our series, most patients responded to glucocorticoids, an immunosuppressant, and/or, in severe cases, rituximab, with functional improvement and normalization of CK typically achieved within 6 months. If these therapies were insufficient or contraindicated (for example, due to active infection or contraindications to glucocorticoids), IVIg would have been considered next. In many countries, the use of IVIg is heavily regulated, or its availability may be limited due to financial or supply constraints. Rituximab may be an alternative in selected cases where the disease course is neither rapidly progressive nor life-threatening, although further research is needed.

The most severely affected patient died within 4 weeks of diagnosis due to anti-HMGCR-IMNM. In cases of rapidly progressive or life-threatening disease, first-line IVIg therapy is justified and recommended.

Many patients experienced residual muscle weakness despite treatment and normalization of CK levels, compared with their pre-disease baseline [1]. Delays in diagnosis and treatment initiation may negatively affect prognosis. Early recognition, discontinuation of statin therapy, and prompt initiation of immunosuppressive treatment are crucial to prevent irreversible muscle damage and improve outcomes. A positive anti-HMGCR-Ab result, as a highly specific marker, virtually confirms the diagnosis, and muscle biopsy may be unnecessary [6, 8, 14].

## Conclusion

Suspicion of statin-associated anti-HMGCR-IMNM should arise in patients with a history of prolonged statin use who present with progressive muscle weakness and elevated CK levels. In our cohort, the most common initial symptom was difficulty walking. Muscle MRI and ENMG, along with testing for other myositis antibodies and muscle biopsy, if necessary, assist in differential diagnosis and assessment of disease severity. The clinical spectrum ranges from mild to fatal disease. Treatment and its intensity must be individualized, taking into account comorbidities and therapy-related risks (e.g. osteoporosis, infection risk, vaccination status, pneumocystis prophylaxis). Early diagnosis and initiation of immunosuppressive therapy, along with rehabilitation and physiotherapy, can prevent irreversible muscle damage and improve prognosis.

## Data Availability

The datasets generated and/or analyzed during the current study are not publicly available due to the Finnish Secondary Use Act governing patient data. Separate applications for research permits and data access permissions must be obtained from the Wellbeing Services County of Southwest Finland for the use of the dataset.
